# Clinical translation and landscape of silver nanoparticles

**DOI:** 10.1007/s13346-024-01716-5

**Published:** 2024-10-08

**Authors:** Manuel Dias, Rui Zhang, Twan Lammers, Roger M. Pallares

**Affiliations:** 1 Institute for Experimental Molecular Imaging, RWTH Aachen University Hospital, 52074 Aachen, Germany; 2 Department of Physics, Faculty of Science, University of Lisbon, Lisboa, 1500-274 Portugal

**Keywords:** Silver nanoparticles, Silver nanoconstructs, Nanomedicine, Clinical trials, Antimicrobial

## Abstract

Despite being clinically used for over a century, the benefits of silver nanoparticles are perennially under debate and dispute. In the last two decades, a revived interest in their therapeutic applications has resulted in a few new formulations transitioning into clinical trials. These metal nanomedicines are used in concrete applications that are defined by the physicochemical and biological features of the silver nanoconstructs, as well as their biodistribution profiles. Examples of these applications are topical antibacterial and antiviral therapies and wound healing, as these avoid concerns regarding the long-term accumulation of the nanomedicines in fenestrated organs after intravenous administration. Here, we discuss the current landscape of silver nanoparticles, and critically analyze the characteristics that endowed their transition and use in clinical settings.

## Graphical abstract

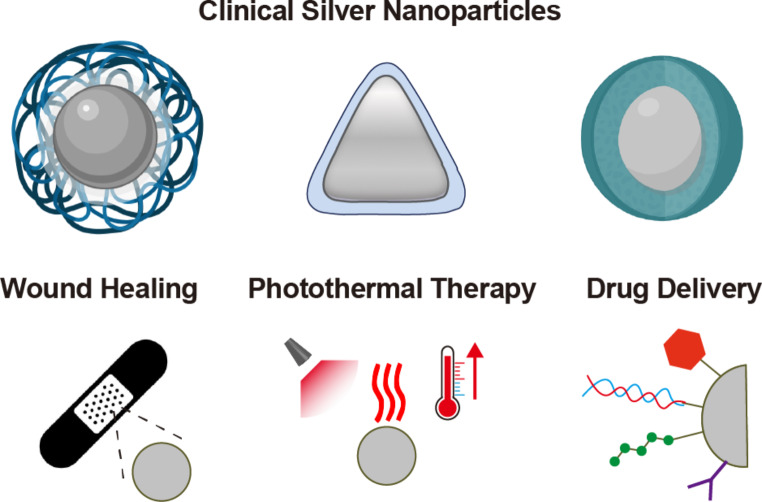

## Introduction

**Fig. 1:**
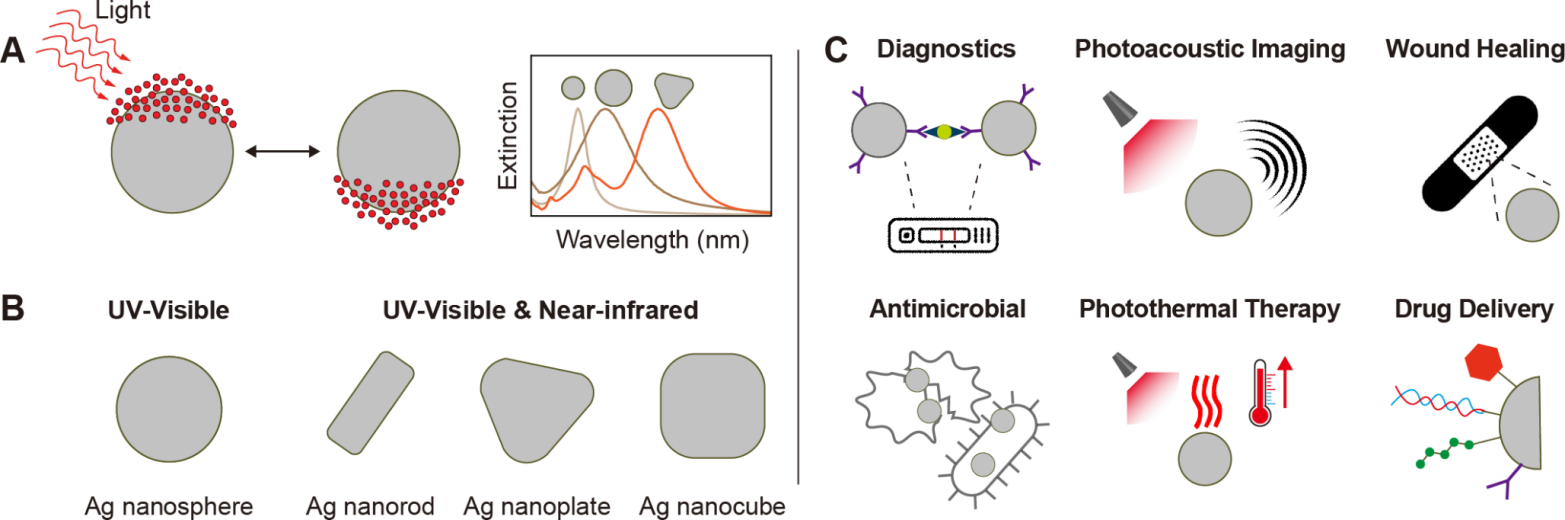
Fundamentals of silver nanoparticles. (**A**) Schematic depiction of localized surface plasmon resonance, and the resulting extinction spectra of different silver nanoparticles. (**B**) Anisotropic silver nanoparticles, such as nanorods, nanoplates and nanocubs, display different optical properties compared to spherical nanoparticles. (**C**) Distinct (pre)clinical applications of silver nanoconstructs

Silver is a metallic element that humans have used for millennia, with applications ranging from currency and jewelry to medicine or (more recently) electronics [1, 2]. In the form of salt, silver has been historically used as a sterilizing agent for wounds [3]. For instance, silver nitrate and silver sulfadiazine have proven effective against gram-positive and gram-negative bacteria [4]. Colloidal silver formulations, such as Collargol and Protargol, were also explored as antiseptic agents at the end of the 19th century and the beginning of the 20th century, but their use rapidly declined with the development of new small-molecule antibiotics [5, 6]. With the emergence of nanotechnology in the last couple of decades, however, new formulations of therapeutic silver have become available, such as silver nanoparticles (AgNPs) [7]. AgNPs display distinct physicochemical properties (Fig. 1) from those observed in ionic and bulk metallic silver [8, 9]. For example, AgNPs possess large surface-to-volume ratios, which result in strong interactions with biological systems and antimicrobial activity [10]. Current evidence suggests that several mechanisms are involved in the antimicrobial effects of AgNPs, including the generation of free radicals and the release of metallic and ionic silver from the particle surface, which induce cell membrane damage and DNA disruption [11]. Nevertheless, the biological activity of AgNPs depends on several factors, such as surface chemistry, size, and shape, which can be controlled during their synthesis [12]. Because of their antimicrobial properties, AgNPs are commonly used as antibacterial agents in industrial, household, and healthcare-related products [13].

Notably, AgNPs also sustain localized surface plasmon resonances, i.e. collective oscillation of their conduction band electrons when interacting with light of specific wavelengths, which yield unique optoelectronic properties [14]. These include strong extinction coefficients in the UV-Vis region of the spectrum and strong electromagnetic fields. The former are commonly exploited in commercial lateral flow assays for the rapid diagnosis of flu and COVID-19, while the latter are employed in analyte identification through surface-enhanced Raman spectroscopy [15–18]. Moreover, AgNPs with anisotropic structures (e.g. plates or rods) or in aggregated form present extinction bands in the near-infrared region of the spectrum, where light penetrates deeper into tissue. Hence, anisotropic and aggregated AgNPs have been (pre)clinically explored for photothermal therapy, where a pathological region is photothermically ablated under laser irradiation [19, 20]. Furthermore, AgNPs can be easily functionalized with ligands to provide new therapeutic attributes and to enhance their stability in biological and harsh environments [21]. As a result, large amounts of preclinical research have explored the use of functionalized AgNPs for drug delivery and gene delivery [22], and immunotherapy [23].

Despite not being as commonly used as gold nanoparticles [24], AgNPs have been quite successful in diagnostics, particularly in the rapid detection of infections via lateral flow assays [25]. Nevertheless, their clinical use in therapy is still rare. Only few therapeutic nanoconstructs have moved to clinical trials, despite the myriad of preclinical studies published yearly reporting novel nanoformulations based on AgNPs. Several factors contribute to this discrepancy between (large) preclinical efforts and (minimal) clinical outputs. For instance, many studies are focused on developing highly sophisticated and exotic nanoformulations that display unique characteristics, rather than solving a real clinical need. Moreover, many preclinical formulations are very complex, with multiple components, making predicting their behavior in vivo and subsequent production and scale-up very difficult. Most new nanoformulations are not competitive enough with current therapeutic standards. Lastly, even though the acute toxicity of AgNPs can be greatly minimized when their surface is functionalized with ligands, such as polyethylene glycol, they tend to display inadequate biodistribution and pharmacokinetics profiles. For instance, after intravenous administration, AgNPs display long-term accumulations in fenestrated organs, such as liver and spleen, which raise concerns regarding their chronic side effects [26, 27]. Considering the above, we describe the current clinical landscape of therapeutic AgNPs in this article. By analyzing the features of silver nanoconstructs that are currently (or have been) explored in the clinic (Table 1), we discuss under what conditions and how AgNPs can provide real therapeutic value for patients.

**Table 1:** Therapeutic silver nanoconstructs in clinical trials

Commercial name	NP formulation	Application	Clinical trial ID^*^	Phase	Status	Results
SilverSol	AgNPs with sizes ranging from 5 to 50 nm and coated with silver oxide, suspension (32 ppm)	Nutraceutical product	NCT01243320	Phase I/II	Completed (2010–2011)	[31]
			NCT01405794	Phase I/II	Completed (2011–2011)	
AgTive	AgNP suspension	Topical antimicrobial	NCT00337714	Phase IV	Completed (2006–2008)	[37]
Sovereign Silver Hydrosol	0.8-nm AgNP suspension (10 ppm)	Topical antimicrobial	NCT02403479	Phase I/II	Completed (2016–2017)	[39]
n/a (in-house produced)	40-nm AgNPs coated with citrate, suspension (0.015 mg/mL)	Topical antimicrobial	n/a	Phase I/II	Completed (2016–2017)	[40]
n/a (in-house produced)	24-nm AgNPs coated with gallic acid, gel (12 ppm)	Topical antimicrobial	NCT02761525	n/a	Completed (2014–2015)	[41]
n/a (not specified)	AgNP cream	Topical antimicrobial	NCT03752424	Phase I	Status unknown (2019–)	n/a
Nowarta110	AgNPs coated with fig extract, suspension	Treatment of plantar warts	NCT02338336	Phase I/II	Completed (2015–2015)	[44]
Argovit	35-nm AgNPs coated with polyvinylpyrrolidone, suspension (1.2% w/w)	Topical antimicrobial for COVID-19 prevention	NCT04894409	n/a	Completed (2020–2020)	[48]
n/a (not specified)	AgNP suspension	Topical antimicrobial to treat COVID-19 infections	NCT04978025	n/a	Status unknown (2020–)	n/a
n/a (not specified)	AgNP gel	Diabetic foot wound healing treatment	NCT04834245	n/a	Completed (2019–2019)	[53]
ASAP Silver Gel	~ 10-nm AgNP gel (24 ppm) with 2.5% benzoyl peroxide	Treatment of severe acne vulgaris	n/a	n/a	Completed (2015–2016)	[54]
n/a (not specified)	AgNP suspension	Photothermal treatment of keratosis pilaris	NCT05666011	n/a	Active, not recruiting yet (2023–)	n/a
SNA-001	Ag nanoplates coated with silica shell, gel	Acne and permanent hair reduction	NCT03039634	n/a	Completed (2016–2018)	[60, 61]

^*^As displayed in clinicaltrials.gov

## Clinical landscape of silver nanoparticles

### Toxicity of oral silver nanoparticles for potential enzymatic therapy

Colloidal silver has become a popular nutraceutical product marketed as an oral health supplement [28]. Despite concerns about their potential toxicological effects, dozens of companies sell numerous colloidal metallic formulations, including AgNPs, as oral supplements with alleged benefits, such as enzymatic activity [29]. Nevertheless, the endogenous behavior, biodistribution, and long-term accumulation of AgNPs in humans remains poorly characterized. In 2010, two phase I/II clinical studies (NCT01243320 and NCT01405794) were initiated at the University of Utah to assess the toxicity of orally administrated commercially available bare AgNPs (with sizes ranging from 5 to 50 nm [30]) coated with a thin layer of silver oxide (SilverSol) through intent-to-treat analyses. Two different doses were explored in 60 healthy volunteers, namely 10 ppm (100 µg silver /day) and 32 ppm (480 µg silver /day), which were administered for up to 14 days. Notably, no significant changes were observed in metabolic, hematologic, and urinary analyses [31]. Moreover, no morphological variations were identified in the lungs, heart or abdominal organs by magnetic resonance imaging. Nevertheless, the trial was limited regarding particle doses and time intervals, and further studies are still required to assess human toxicity thresholds of orally administered AgNPs accurately.

### Silver nanoparticles as antimicrobial agents

Collargol and Protargol (also known as silver albuminate) were the first successful silver nanoformulations to be developed for antibiotic applications [32]. They were developed between 1894 and 1897 as less irritating and more effective alternatives to silver nitrate in treating gonorrhea. Collargol was made of 10-nm AgNPs, while Protargol was comprised of 1-nm silver cores where the silver was in metallic and/or oxidized state [33, 34]. Furthermore, their clinical use later expanded to other treatments, such as antirheumatic therapy [35]. They were a big commercial success and were widely adopted until the development of new small-molecule antibiotics, when their use rapidly declined. Nowadays, both Collargol and Protargol are still being commercialized as over-the-counter liquid medications against nose and ear infections in the USA and Europe.

#### Silver nanoparticles in central venous catheters to protect against infections

Bloodstream infections are a major threat in intensive care units (ICU), since they are common and have a 35% mortality rate [36]. Central venous lines further increase the risk of bacterial infections, and as a result, around 25% of all ICU bloodstream infections are associated with using catheters. Thus, coating or impregnating central venous lines with antimicrobial materials has been proposed to prevent infections. In 2006, a pioneer randomized multicenter clinical trial (NCT00337714) assessed the ability of catheters impregnated with a commercial AgNP solution (AgTive) to prevent infections in ICU patients. The patients were divided into two groups, as they were treated with either standard triple-lumen non-modified catheters or triple-lumen catheters impregnated with AgTive. The clinical study found no significant differences in the colonization rates (33% and 30% in the Ag-coated and non-coated catheter groups, respectively) or in the colonization and related bloodstream infections incidence rates (3.4 infections per 1000 catheter days in both groups) [37]. The study concluded that in critically sick ICU patients, the coating of catheters with AgTive had no significant impact on colonization, infection incidence, and mortality rates. As a result, the AgTive coating was not recommended.

#### Topical silver nanoparticles as antibacterial agents

Rhinosinusitis is a highly prevalent disease (occurring in up to 30% of the population), where the mucous membranes in the sinuses get inflamed [36]. Rhinosinusitis is usually triggered by an earlier respiratory tract disorder, such as bacterial or viral infections. In 12.5% of the people, the disease becomes chronic, with inflammation of the sinuses lasting longer than 12 weeks, and in at least 10% of those patients, medical and surgical treatments do not resolve the symptoms, and the disease becomes refractory [38]. The formation of rhinosinusitis-associated biofilms is believed to contribute to the chronification of the pathology. Nevertheless, increasing (pre)clinical evidence has shown that AgNPs may effectively reduce biofilms and treat numerous microbial infections. As a result, four clinical trials assessing the use of AgNPs as topical antibacterial agents have been initiated in the last decade.

The first clinical trial (phase I/II) started in 2016 (NCT02403479) at the Lawson Health Research Institute (Canada), and explored the use of AgNP hydrosols for the treatment of patients with refractory chronic rhinosinusitis. Twenty randomized patients were treated with AgNP and saline solutions following a crossover methodology. A commercially available AgNP solution (Sovereign Silver Hydrosol) was used as a silver topical therapeutic. Although no adverse effects attributable to the administration of the AgNPs were reported, no subjective or objective benefits were identified in comparison to the control group [39]. As a result, topical administration of AgNPs was noted to be safe but not therapeutically superior to commonly used nasal sprays.

A second phase I/II clinical study conducted at the University of Adelaide (Australia) identified that despite displaying antibacterial activity during the treatment of recalcitrant chronic rhinosinusitis, sinus rinses with 40-nm AgNPs were not superior to culture-derived oral antibiotics [40]. This was a prospective, open-label, single-blinded pilot study with patients who had previously undergone endoscopic sinus surgery but still displayed sinus infection signs and positive bacterial cultures. Sinus rinses with 0.015 mg/mL AgNPs twice daily for ten days demonstrated an adequate safety profile without significant adverse events. Both treatment groups, namely AgNPs and oral antibiotics, showed similar improvements in symptoms and endoscopic scores. Thus, this study concluded that AgNP rinses (0.015 mg/mL, twice daily) for ten days were safe and therapeutically active but not superior to culture-directed oral antibiotics.

Beyond rhinosinusitis, two clinical trials have assessed the use of silver colloids in the topical treatment of bacterial infections. In a study completed (NCT02761525) at the Universidad Autónoma de San Luis Potosí (Mexico), AgNPs were explored for treating oral pathogens in patients intubated with mechanical ventilators. The study determined that gels containing 12 ppm of 24-nm AgNPs (produced in house) and applied in the oral mucosa surface of mechanically ventilated patients could reduce up to 9-fold the formation of pathogenic microbial colonies in comparison to the same gel without silver colloids [41]. In a second phase-I study (NCT03752424) at the Al-Azhar University (Egypt), a vanishing cream containing AgNPs is being explored to treat dermal bacterial and fungal infections.

#### Silver nanoparticles in the treatment of virus-caused plantar warts

**Fig. 2:**
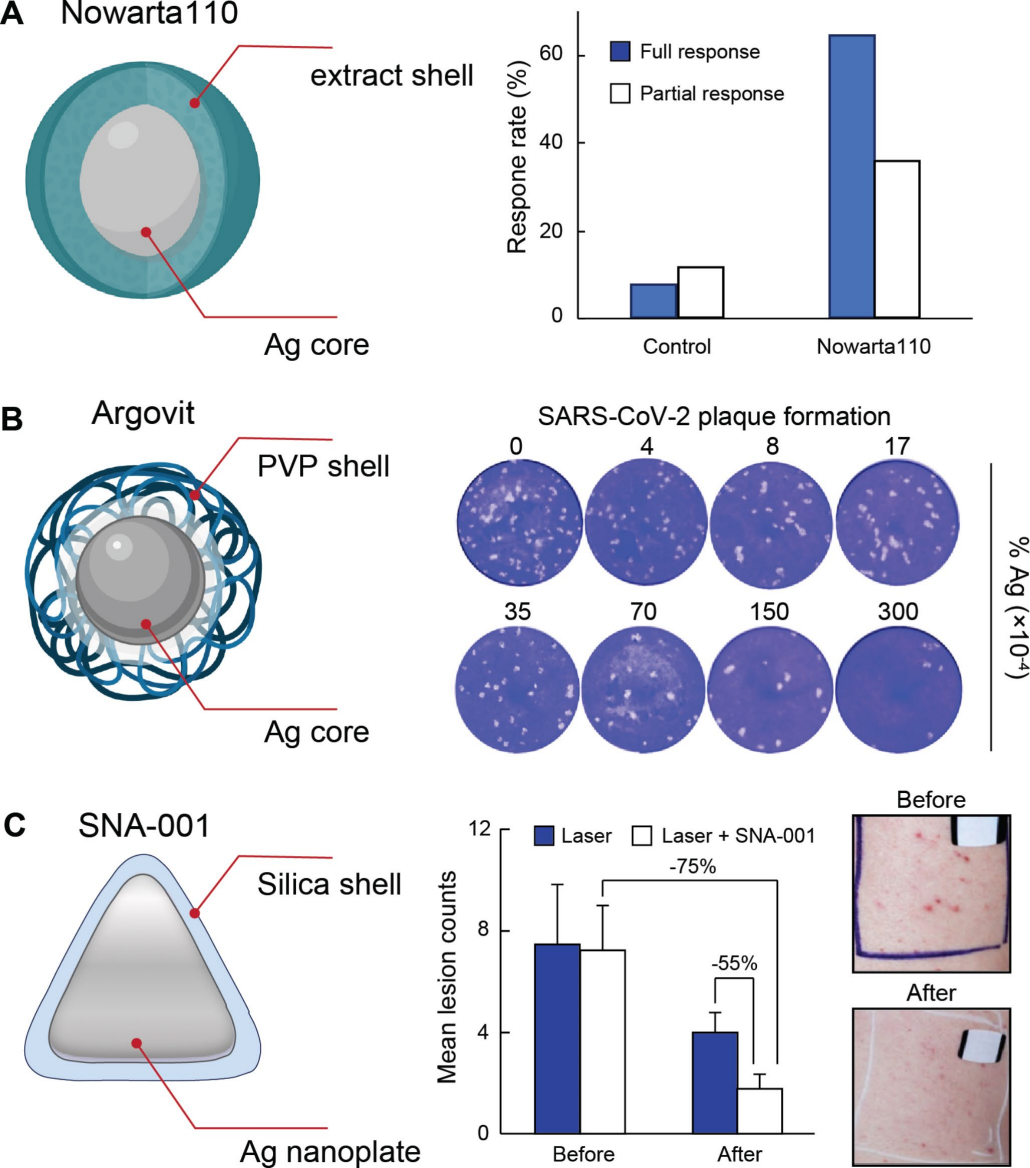
Representative therapeutic silver nanoconstructs being explored in clinical trials. (**A**) Schematic representation of Nowarta110 and its response rates against plantar warts as reported in Ref. 44. The nanoparticle schematic has been adapted with permission of BioRender (2024). The column figure has been obtained by plotting the data reported in Ref. 44. (**B**) Schematic depiction of Argovit and representative SARS-CoV-2 plaque formation in the presence of different content of the silver nanoconstruct. The nanoparticle schematic has been adapted with permission of BioRender (2024). The SARS-CoV-2 plaque data has been adapted with permission of Ref. 48. Copyright 2021 Almanza-Reyes et al. (**C**) Schematic representation of SNA-001 and its therapeutic performance against moderate and severe acne. Adapted with permission of Ref. 61. Copyright 2017 Sienna Biopharmaceuticals

Plantar warts are benign epithelial tumors on the bottom of the feet [42]. These rough skin growths are caused by the human papillomavirus, which infects compromised skin through direct exposure. The severity and magnitude of plantar warts range from discomfort to severe pain, and their treatment remains a challenge. In 2015, a phase-I/II study (NCT02338336) was conducted by Nowarta Biopharma to evaluate the safety and efficacy of Nowarta110 in treating patients with plantar warts. Nowarta110 is a formulation containing fig extract as a main therapeutic component and uses AgNPs for dermal penetration and supplementary antimicrobial activity. It is worth noting that fig tree latex (a milky extract of the leaves and fruits of fig trees) is known to display therapeutic activity against human papillomavirus [43]. The study included 54 patients with plantar warts, randomly separated into a placebo group and a group receiving topical Nowarta110. Notably, all 28 patients treated with Nowarta110 displayed positive responses (Fig. 2A), with 18 out of the 28 patients presenting complete clearance of their warts and the remaining ten patients showing between 20 and 80% decrease in their lesion dimensions [44]. In the placebo group, however, only 5 out of the 26 patients responded positively to the therapy, with 2 presenting full recovery of their warts and 3 displaying between 10 and 35% decrease in their wart dimensions. In addition to its therapeutic efficacy, Nowarta110 showed very low side effects, since only one minor pain event was reported. Hence, topical Nowarta110 proved to be well tolerated and safe, as well as highly effective against recurrent and refractory plantar warts. The therapeutic efficacy of Nowarta110 will be further evaluated in a phase III clinical trial that is scheduled for late 2024.

#### Silver nanoparticles to prevent COVID-19 infections

The COVID-19 outbreak collapsed the healthcare systems of hundreds of countries in just a few months, as the SARS-CoV-2 virus was highly contagious and transmitted by symptomatic and asymptomatic carriers alike. In the middle of the pandemic, a prospective randomized study (NCT04894409) assessed the prevention of SARS-CoV-2 infection in health workers with a high risk of exposure by using Argovit, a commercially available silver-based antiviral formulation. Argovit is a suspension of 35-nm AgNPs coated with polyvinylpyrrolidone (PVP) [45] and has been primarily explored for veterinary applications in Russia [46, 47]. 231 participants were instructed to perform nose rinses and mouthwash with either conventional solutions (control group) or Argovit (1% w/w silver) solutions for 9 weeks (Fig. 2B). The incidence of SARS-CoV-2 was significantly lower in the AgNP group than in the control group (1.8% and 28.2%, respectively), representing an 84.8% efficiency of the silver-based treatment [48]. Hence, this clinical study concluded that nasal and mouth rinses with AgNP solutions significantly prevented SARS-CoV-2 infection in staff exposed to patients diagnosed with COVID-19.

In another clinical study in the Hôpital Universitaire Sahloul (Tunisia), AgNPs are being studied for the treatment (rather than prevention) of COVID-19. In this study (NCT04978025), SARS-CoV-2 infected patients are treated with AgNPs by inhalation (5-mL nebulized dose) or oral administration (30-mL solution dose) thrice a day for 5 days. In the control groups, patients receive a placebo solution (orally or by inhalation). The clinical status of the patients is evaluated on day 10, and adverse events on the first month.

### Silver nanoparticles for wound healing

Silver salts have been historically used in wound care because of their strong antimicrobial, anti-inflammatory and antioxidative activities [49]. In the last two decades, an increasing body of literature has reported AgNPs being even more effective than their salt counterparts [50]. Nevertheless, silver salts and AgNPs also have downsides, as they can potentially impair healing by disrupting fibroblasts and keratinocytes [51]. Because the existing literature on the application of silver salts and AgNPs in wound healing is rather heterogeneous, it has been challenging to develop treatment guidelines. Recently, a meta-review identified that AgNP-based dressings are beneficial in infected wound treatments and burn healing during the first days (up to a few weeks) post-injury, after which they should be replaced by other therapies, such as pressured treatments [52]. However, treatments based on silver provide no benefits for clean wounds and closed surgical incisions.

In 2019, a clinical trial (NCT04834245) was conducted to evaluate the performance of a hydrogel dressing based on AgNPs to treat diabetic foot ulcers in type-2 diabetes patients. The AgNP-based treatment improved ulcer healing by increasing wound contraction and the rate of re-epithelialization [53]. Hence, in the third week of the treatment, the ulcers treated with the AgNP-based dressing had shrunk down to 15% of their original size, compared to 33% in the case of the wounds treated with conventional dressings. Nevertheless, it is worth noting that the number of patients in this study was relatively small (sixty), and a larger cohort of patients may be necessary to further confirm these findings. Furthermore, other factors, such as costs and treatment frequency, need to be considered when assessing the benefits of the new therapeutic protocol. While AgNP treatments were more costly, they could be applied once every three days, in comparison to conventional dressings that had to be reapplied daily.

The antimicrobial and anti-inflammatory activities of AgNPs have also been exploited against severe acne vulgaris. Despite being commonly treated with comedolytic agents and antibiotics, alternative therapeutic agents for severe acne are being explored due to increasing bacterial resistance to topical antimicrobials. In a double-blinded, randomized-controlled study, sixty-four patients with severe acne were treated with benzoyl peroxide and ASAP Silver Gel (24 ppm AgNP gel) or clindamycin (antibiotic) gel. The study confirmed the good safety profile of the AgNP treatment, which was demonstrated to be as effective as the antibiotic treatment [54].

### Silver nanoparticles as photothermal agents for skin conditions

Keratosis pilaris (also known as chicken skin) is a dermatological disorder where patches of rough pimples (or bumps) appear on the skin due to the plugging of hair follicles by dead skin cells [55]. This skin condition is not contagious and preferentially develops on the thighs, upper arms, buttocks, and cheeks. Currently, there is no cure for the pathology, however, some treatments can limit its progression and worsening. The Lumenis M22 Intense Pulsed Light is a handheld device that irradiates pulsed laser light to treat various skin conditions. Because AgNPs display strong extinction coefficients, due to their localized surface plasmon resonances, and efficient photothermal capabilities, they have been extensively used at preclinical level to photothermically ablate pathological tissue under laser irradiation [56, 57]. In 2023, a pilot study (NCT05666011) was initiated (the patients have yet to be recruited) to explore whether the dermatological benefits of pulsed laser with the Lumenis M22 device can be further improved with AgNPs during the treatment of keratosis pilaris. For the AgNP-enhanced therapy, the patients will be treated with a 0.5 mL AgNP suspension administered with an infusion paddle for 5 min before the laser irradiation. Patients will receive three cycles of treatment spaced four to six weeks apart, and will be evaluated at 1 and 3 months.

SNA-001 is a topical gel suspension of Ag nanoplates with strong absorbance coefficients in the near-infrared. Because the optical properties of AgNPs depend on their morphology, the Ag nanoplate sizes are tuned to the wavelengths of common dermatological lasers (755 nm, 810 nm and 1064 nm). SNA-001 particles do not penetrate the epithelial layer, and are used as photothermal agent against sebaceous glands and hair follicles for acne and hair removal therapies [58]. For instance, in an evaluator-blinded controlled study (NCT03039634), SNA-001 combined with 810-nm diode laser showed − 29.4% hair removal in comparison to the control treatment (gel and laser) that only yielded − 10.4% hair removal. Furthermore, the SNA-001 treatment could remove both lighter and darker hair, in contrast to the laser treatment that only reduced darker hair due to the presence of greater amounts of melanin (natural pigment) [59]. SNA-001 also showed improved efficacy in the treatment of moderate and severe acne by photoablating sebaceous glands (Fig. 2C) and acceptable safety in an interim analysis [60, 61]. The study, however, was limited by the small sample size (ten patients) and the use of different laser settings.

## Conclusions

There has been a renewed interest in therapeutic AgNPs in the last two decades. Nevertheless, despite the extensive preclinical efforts that every year report new silver nanoconstructs for a wide range of medical applications, the clinical use of AgNPs is limited to very specific therapies, such as topical antibacterial and antiviral treatments, and wound healing. These applications benefit from the antimicrobial and anti-inflammatory properties of colloidal silver. Furthermore, AgNPs are applied in topical therapies, as these minimize concerns regarding (potential) chronic effects due to the long-term accumulation of the colloids in fenestrated organs after intravenous administration. AgNPs being explored in clinical trials also have simple compositions, as their in vivo behavior is easier to study and predict, and their production is easier to scale up. To what extent AgNP-based therapies can surpass gold standard treatments remains unclear at the moment, since only a few studies have shown the benefits of the former. Altogether, after more than a century of clinical use, the therapeutic benefits of AgNPs remain a source of debate, with some clinical evidence showing promising results in specific niche treatments, where AgNPs, with their unique biological and physicochemical properties, outperform other available formulations.
